# Structural Evolution of Printed Ternary Magnetic Hybrid Thin Films Containing Soft and Hard Magnetic Nanoparticles for Coupled Composites

**DOI:** 10.1021/acsami.5c16986

**Published:** 2025-11-25

**Authors:** Christopher R. Everett, Guangjiu Pan, Manuel A. Reus, David P. Kosbahn, Aidin Lak, Frank Hartmann, Martin Bitsch, Markus Gallei, Matthias Opel, Matthias Schwartzkopf, Peter Müller-Buschbaum

**Affiliations:** † 9184 Technical University of Munich, TUM School of Natural Sciences, Department of Physics, Chair for Functional Materials, James-Franck-Str. 1, Garching 85748, Germany; ‡ Institute for Electrical Measurement Science and Fundamental Electrical Engineering and Laboratory for Emerging Nanometrology (LENA), 9183 TU Braunschweig, Hans-Sommer-Str. 66, Braunschweig 38106, Germany; § Chair in Polymer Chemistry, 9379 Saarland University, Campus C4 2, Saarbrücken 66123, Germany; ∥ Saarene, Saarland Center for Energy Materials and Sustainability, Campus C4 2, Saarbrücken 66123, Germany; ⊥ Walther-Meissner-Institut, 226241 Bayerische Akademie der Wissenschaften, Walther-Meissner-Str. 8, Garching 85748, Germany; # 28332 Deutsches Elektronen-Synchrotron (DESY), Notkestr. 85, Hamburg 22607, Germany

**Keywords:** ternary hybrid films, magnetic nanoparticles, printing, GISAXS, dipolar interaction

## Abstract

Diblock copolymer thin films templating two types of magnetic nanoparticles are ternary nanocomposites that can show tunable magnetic behavior depending on the size and magnetic properties of the nanoparticles. In the case of utilizing both soft and hard magnetic nanoparticles, the ternary films become interesting for applications in permanent magnets and microwave devices. In this work, ternary hybrid thin films composed of the diblock copolymer polystyrene-*block*-poly­(methyl methacrylate) (PS*-b*-PMMA), cobalt ferrite (CoFe_2_O_4_, *d* = 21.7 ± 12.2 nm) nanoparticles, and nickel (Ni, *d* = 46 ± 10 nm) nanoparticles are fabricated from solution in a slot-die printing process. The film morphology evolution is tracked *in situ* by grazing-incidence small-angle X-ray scattering (GISAXS). For comparison, a binary hybrid film with only PS-*b*-PMMA and CoFe_2_O_4_ nanoparticles and a pure PS-*b*-PMMA film are also investigated. All films show similar kinetics during film formation, where the wet film undergoes solvent evaporation followed by rapid microphase separation and coalescence into the final dry film. Complementary atomic force microscopy (AFM) measurements reveal the as-printed surface morphology of the polymer nanocomposites. To probe the magnetic behavior of the hybrid thin films, a superconducting quantum interference device (SQUID) magnetometer is used to measure the magnetic response in both the in-plane direction and out-of-plane direction. The ternary film shows a single-phase hysteresis loop at 300 K that evolves into a two-phase hysteresis as the temperature is decreased, as the soft and hard magnetic phases switch individually. Compared to the binary film, the ternary film shows increased coercivity over the measured temperature range due to dipolar coupling between the NPs in the system. Thus, the ternary film demonstrates the potential for utilizing dipolar interactions in the fabrication of coupled composites, allowing for the tuning of magnetic behavior without the need for complex material synthesis.

## Graphical abstract

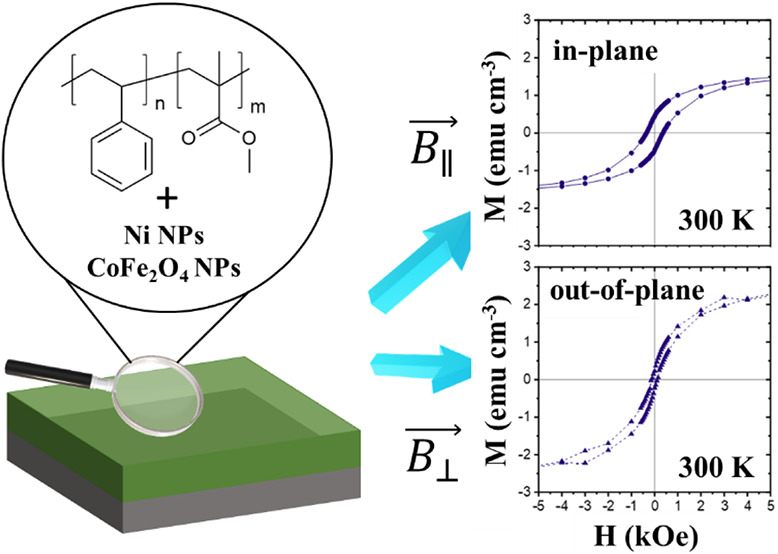

## 1 Introduction

Composites containing both hard and soft magnetic materials are unique systems that advantageously combine the magnetic properties of the constituent materials. Through the combination of the high saturation magnetization of the soft magnetic material with the large coercivity of the hard magnetic material, enhanced magnetic properties can be achieved. Furthermore, if exchange coupling between the components is achieved, so-called exchange spring magnets (ESMs) can be fabricated, where the maximum energy product of the material is increased. This behavior can be observed in a rather wide, square-shaped hysteresis loop. Such soft/hard magnetic composites are useful for a variety of applications, including permanent magnets, microwave devices, data-storage systems, and magnetic sensors.^1–3^

As such magnetic interactions take place on the nanometer scale; magnetic nanoparticles (NPs), instead of bulk materials, are now widely used in research on soft/hard magnetic composites, ESMs, and permanent magnets.^4–8^ Magnetic NPs can show unique size-dependent properties compared to the bulk. In the case of ferromagnetic or ferrimagnetic NPs, as the size of the NPs is decreased, the number of magnetic domains in the individual particles is reduced.^9,10^ This corresponds to an increase in the coercivity, H_c_, of the particles that reaches a maximum at the point where the particles transition from having multiple domains to having a single magnetic domain. Thus, through careful control of the NP synthesis, it is possible to tailor the magnetic properties of NPs in order to achieve the desired magnetic behavior.^11–13^

Utilizing magnetic NPs, previous studies have focused on the synthesis of magnetic NP coassemblies and core/shell NPs as soft/hard magnetic nanocomposites. Using binary assemblies of FePt NPs and Fe_3_O_4_ NPs, Zeng et al. fabricated soft/hard magnetic nanocomposites where the effectiveness of the exchange coupling depended strongly on the size of the soft magnetic phase.^14^ Chen et al. fabricated ordered superlattices of CoFe_2_O_4_ and Fe_3_O_4_ nanocrystals through drop-casting and subsequent evaporation. The nanocomposites demonstrated exchange coupling upon thermal annealing.^15^ In the case of core/shell NPs, Zeng et al. used a coprecipitation method to synthesize CoFe_2_O_4_/Fe_3_O_4_ nanocomposites and showed that these composites exhibited enhanced magnetic properties compared to a simple mixture of CoFe_2_O_4_ NPs and Fe_3_O_4_ NPs.^16^ However, it has been shown that long-range dipolar interactions between the soft and hard magnetic NPs can significantly affect the magnetic behavior of a nanocomposite, particularly in systems where exchange coupling may not be prevalent.^17–20^ This consideration is important with respect to dilute NP systems where the NPs or NP structures may not be in immediate contact with one another. Murry et al. investigated binary assemblies of Fe_3_O_4_ nanocrystals of two distinct sizes in addition to binary assemblies of Fe_3_O_4_ and FePt nanocrystals. In both cases, the assemblies exhibit interparticle dipolar interactions, as observed through their collective magnetic behavior.^17^ Furthermore, Le et al. demonstrated constructive dipolar coupling arising from ordered stacks of mixed hard and soft FePt NPs in a mesoporous silica matrix.^18^ In such studies on magnetic NP nanocomposites, the complexity of material synthesis and the orientation of the NPs within the composite can often create challenges.

For a wide variety of applications, diblock copolymers (DBCs) are used as scaffolds and templates for NPs and magnetic NPs.^21–30^ DBCs can undergo microphase separation upon deposition out of solution to form thin films with ordered nanostructures such as spheres, cylinders, gyroids, and lamellae.^31–35^ Furthermore, by increasing the molecular weight of the DBC, the size of the nanostructures can be increased. Previous work has shown that for ultrahigh molecular weight (UHMW) DBCs, where *M*_n_ > 5 × 10^5^ g mol^–1^, domain sizes with *d* > 80 nm can be realized.^36–41^ This makes UHMW DBCs versatile matrices that can also be suitable for hosting large NPs.^42^ In contrast to homopolymers, DBCs provide another advantage in that the localization of the NPs can be tailored so that a particular NP species localizes preferentially to one of the polymer blocks. This behavior can be realized through surface modification of the NPs and/or through careful control of the NP size.^43–47^ NP surface functionalization can reduce the likelihood of the expulsion of NPs from the DBC matrix arising from entropy losses due to the confinement of the polymer chains.

In this study, soft/hard magnetic nanocomposites are fabricated utilizing nickel (Ni) NPs, CoFe_2_O_4_ NPs, and a UHMW PS-*b*-PMMA DBC as a scaffold. The films are prepared via a slot-die printing process from a solution. Using *in situ* grazing-incidence X-ray scattering (GISAXS), the influence of the magnetic NPs on the film morphology evolution is examined. The local surface properties of the prepared films are investigated with atomic force microscopy (AFM). Finally, the in-plane and out-of-plane magnetic properties of the hybrid thin films are probed with a superconducting quantum interference device (SQUID) magnetometer. For each orientation, the magnetic properties are probed at five different temperatures, and the interaction between the magnetically soft Ni NPs and the magnetically hard CoFe_2_O_4_ NPs is examined. Importantly, while the two-phase magnetic behavior of the composites demonstrates the absence of exchange coupling, the increase in coercivity of the soft/hard composite indicates the presence of dipolar interactions. Thus, this work gives further insight into the synthesis of soft/hard magnetic nanocomposites without difficult synthesis steps and motivates further investigation of such flexible materials to create magnetically coupled composites that can be operated over a wide temperature range.

## 2 Experimental Section

### 2.1 Materials

A symmetric amphiphilic polystyrene-*block*-poly­(methyl methacrylate) (PS-*b*-PMMA) DBC was synthesized by anionic polymerization in a similar process as described in a previous publication.^48^ The PS-*b*-PMMA had an average molar mass (*M*_n_) of 867 kg mol^–1^, a polydispersity index of 1.10, and a PMMA volume fraction (φ_PMMA_) of 55.4%. Nickel NPs (Ni, *d*_TEM_ = 46 ± 10 nm) functionalized with PMMA ligands were synthesized by a chemical precipitation route as detailed in a previous publication.^49^ Cobalt ferrite NPs (CoFe_2_O_4_, *d* = 21.7 ± 12.2 nm) coated in oleic acid ligands were synthesized via thermal decomposition and suspended in chloroform.^50^

### 2.2 PS-*b*-PMMA/Nanoparticle Film Preparation

To prepare the thin films, toluene (Sigma-Aldrich) was used to dissolve the PS-*b*-PMMA to create solutions with a concentration of 10 mg mL^–1^. The solutions were left overnight on a shaker to ensure complete dissolution of the polymer. In addition to the pure DBC film without NPs, a binary hybrid film containing the DBC and CoFe_2_O_4_ NPs and a ternary film containing the DBC and both CoFe_2_O_4_ NPs and Ni NPs were chosen for investigation. Thus, with respect to PS-*b*-PMMA, the three selected weight ratios (wt %:wt %) of Ni NPs to CoFe_2_O_4_ NPs were 0:0, 0:2, and 2:2. For the binary and ternary films, the NPs were added 1 h before the *in situ* GISAXS investigation to the polymer solutions. The films were prepared on precleaned Si substrates using a meniscus-guided slot-die printing procedure, which was optimized for reproducibility through pretests in the laboratory at TUM and is detailed in our previous publication.^49^

### 2.3 *In Situ* GISAXS Analysis

*In situ* GISAXS experiments were carried out on one film from each investigated sample system (0:0, 0:2, 2:2) at the P03 (MiNaXS) beamline at DESY (Hamburg, Germany).^51^ A detailed description of the experimental parameters can be found in the Supporting Information.

### 2.4 *Ex Situ* Analysis

After deposition, the film surface morphology was examined using an AFM (CoreAFM, Nanosurf) in tapping mode. The measurements were conducted in air with a monolithic silicon cantilever coated in aluminum (TAP190Al-G, BudgetSensors). To investigate the magnetic behavior of the binary and ternary hybrid films, a SQUID magnetometer (MPMS XL-7, Quantum Design) in direct current mode was used to probe the films over a range of temperatures (5, 50, 100, 200, and 300 K). The samples were measured in two different configurations to obtain both the magnetizations in the film plane and out of the film plane, with the applied external magnetic field (−70,000 to 70,000 Oe) in the in-plane scenario being parallel to the sample surface, while in the out-of-plane scenario, the applied external magnetic field is applied perpendicular to the sample surface.

## 3 Results and Discussion

### 3.1 Surface Morphology of Printed Films

Utilizing AFM, the surface morphology of the films after printing is investigated. As predicted by the self-consistent mean-field theory, and due to the high segregation strength between the PS and PMMA blocks, the UHMW DBC forms lamellar PS and PMMA domains in the thin films. The AFM topography image of the pure DBC film without NPs is shown in Figure 1a. The bright domains, having an increased height, are assigned to PMMA, while the dark domains are assigned to PS.^52,53^ Due to the rapid nature of the microphase separation during film formation, leading to a “freezing” of the film morphology before significant chain reorganization can occur, the domains of the DBC appear as short, worm-like structures. Upon addition of 2 wt % CoFe_2_O_4_ NPs, the surface morphology of the binary hybrid thin film does not change significantly. Similarly, as seen in Figure 1c, the further addition of 2 wt % Ni NPs in the ternary hybrid film preserves the surface morphology of the thin film. Thus, the DBC film morphology appears to be able to incorporate small quantities of NPs without significant disruption. Furthermore, no NPs are readily observed on the film surface, suggesting that the NPs were successfully embedded inside the hybrid thin films.

**Figure 1:**
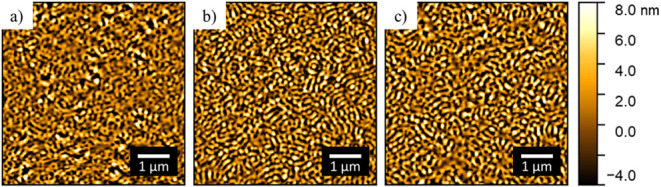
AFM topography images (5 μm × 5 μm) of the as-printed thin films with varying NP concentrations: (a) pure DBC film with no NPs, (b) binary hybrid film with 2 wt % CoFe_2_O_4_ NPs, (c) ternary hybrid film with 2 wt % CoFe_2_O_4_ NPs and 2 wt % Ni NPs.

### 3.2 Morphology Evolution during Printing

*In situ* GISAXS measurements are used to track the morphology of the investigated films *in situ* in order to determine the influence of the NPs on the film formation process. Film formation occurs after deposition of the polymer/NP solution onto a clean silicon substrate, a process which is described in detail in the experimental section. As the print head is moved in one direction to deposit the film, the sample is scanned with the X-rays in the opposite direction. Thus, the starting point of the film formation process, 0 s, is defined as the position where the print head crosses the path of the X-ray.

For the DBC film without NPs, representative 2D GISAXS data can be seen in Figure S1. Immediately after deposition, a strong scattering signal is observed from the solution. As time increases, the evolution of the typical DBC film morphology is found with the sudden appearance of scattering features at large *q*_*y*_ values. After this, the scattering features remain constant.

From the 2D GISAXS data, horizontal line cuts are taken and modeled in the framework of the distorted-wave Born approximation (DWBA), local monodisperse approximation (LMA), and effective interface approximation (EIA).^54,55^ The data modeling was described in detail in our previous publication.^49^ The horizontal line cuts are taken at the Yoneda peak position (i.e., at the critical angles of the two polymer constituents).^56^ This is possible as the polymer components have, at an incidence angle of α_i_ = 0.4°, different critical angles of α_c,PS_ = 0.102° and α_c,PMMA_ = 0.111°. Figure 2a shows the extracted data, black points, and the respective fits, red curves, corresponding to the time at which the data were collected. As time increases, two distinctive polymer domain peaks develop, as noted by the dark gray and light gray arrows. The center-to-center distance, D, and radius, R, information extracted from the fits with respect to time can be seen in Figure 2b for the large polymer domain, D_1_ and R_1_, and the small polymer domain, D_2_ and R_2_. The film formation process can be divided into four stages. In the first stage, 0 s < *t* < 25 s, no scattering information from the polymer domains is observed, and the scattering information is dominated by scattering from solution in addition to a noticeable background scattering at large *q*_*y*_ values of approximately 0.1 nm^–1^ < *q*_*y*_ < 1 nm^–1^. In the second stage, 25 s < *t* < 35 s, only information about the small polymer domain is observed and characterized by a cylindrical domain with a radius of (25 ± 5) nm. This length is attributed to the size of the polymer domains themselves and shows no higher ordering. In the third stage, 35 s < *t* < 42 s, a rapid coalescence and microphase separation occur with the appearance and quick consolidation of both the large and small polymer domains. The large domains, show a decrease in the size of the domains from (56 ± 8) nm to (47 ± 3) nm and a corresponding decrease in the center-to-center distance from (140 ± 10) nm to (130 ± 10) nm while the small domains decrease in size from (25 ± 5) nm to (16 ± 3) nm and the center-to-center distance of the small domains decrease as well from (90 ± 10) nm to (70 ± 10) nm. As the solvent leaves the film, the domains shrink and move closer together. The time it takes for rapid coalescence and microphase separation to occur is defined as the rate of self-assembly and occurs within 7 s for the DBC film without NPs. In the final stage, *t* > 42 s, the film morphology remains constant, and no significant changes in the domain size or domain ordering are observed, resulting in a stable film morphology. The observed four stages of film formation (I–IV) are ascribed to the wet film, solvent evaporation, a rapid microphase separation, and the final dry film. The film formation follows a similar process to the one described in our previous publication, where the evaporation of the toluene solvent drives the coalescence and microphase separation.^49^

**Figure 2:**
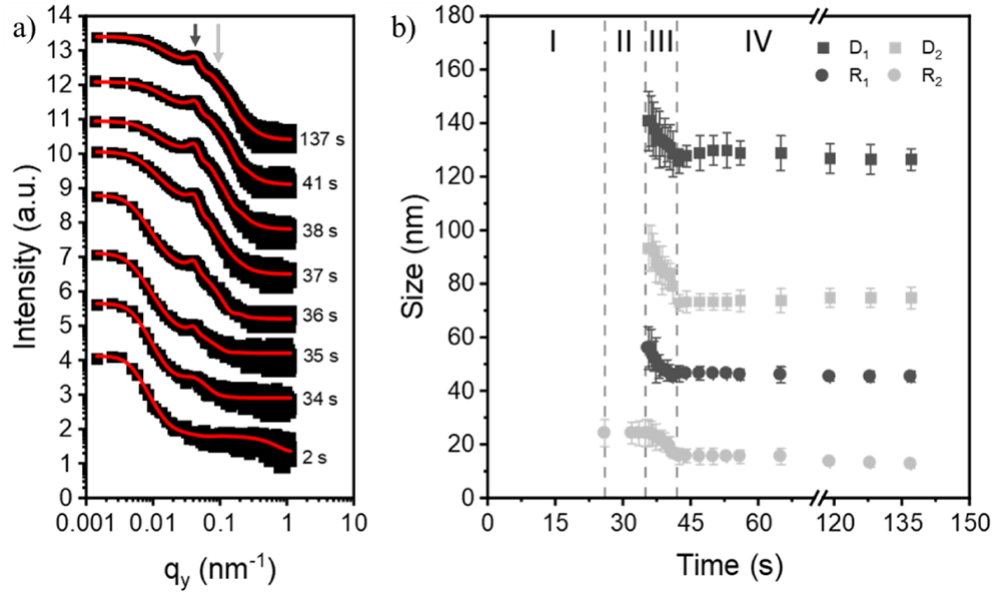
(a) Selected line cuts for the pure PS-*b*-PMMA film with no NPs taken from the 2D GISAXS data. The curves are shifted vertically along the *y*-axis according to the time of data collection during the slot-die printing process. The data are modeled within the framework of DWBA, LMA, and EIA, and the fits, red lines, are overlaid on the data. Two characteristic peaks corresponding to the polymer domains, one large domain and one small domain, evolve during film fabrication (dark gray and light gray arrows). (b) Radius and distance information on the large polymer domains (D_1_ and R_1_) and small polymer domains (D_2_ and R_2_) taken from the fits. The polymer domains are assumed to be cylindrical. Film formation can be divided into four stages (I–IV): wet film, solvent evaporation, coalescence and microphase separation, and the dry film.

Using the DBC film without NPs as a reference, the film morphology evolution of the binary hybrid film containing 2 wt % CoFe_2_O_4_ NPs is investigated. Representative 2D GISAXS data can be seen in Figure S2. With the addition of the CoFe_2_O_4_ NPs, large wing-like scattering features appear at large q_*y*_ values. In addition, the oscillations near the Yoneda region corresponding to a correlated roughness between the substrate and the surface of the thin film are smeared out. This finding implies that the incorporation of the NPs influences the film deposition. The corresponding horizontal line cuts and fits can be seen in Figure 3a. Similarly, to the film without NPs, two polymer domain peaks appear and increase in intensity (Figure 4a). The peaks are highlighted with a dark orange and light orange arrow, indicating the large and small domain peaks. Furthermore, a peak near 0.2 nm^–1^ is observed. This peak corresponds to the CoFe_2_O_4_ NPs, where the NPs are modeled as spheres. To account for potential NP aggregates, the center-to-center distance is twice the radius. As the NPs have a constant size and the peak position only shows changes in intensity, only the radius and center-to-center information on the two polymer domain peaks are shown in Figure 3b. The binary hybrid film also shows four stages of film formation: wet film (0 s < *t* < 26 s), solvent evaporation (26 s < *t* < 35 s), microphase separation and coalescence (35 s < *t* < 41 s), and the dry film (*t* > 41 s).

**Figure 3:**
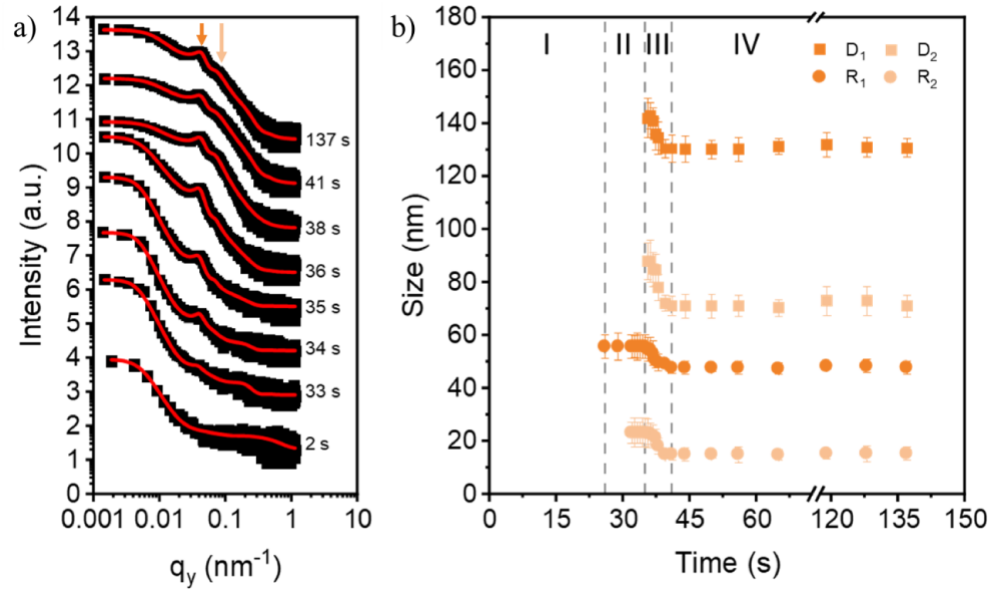
(a) Selected line cuts for the pure PS-*b*-PMMA film containing 2 wt % CoFe_2_O_4_ NPs taken from the 2D GISAXS data. The curves are shifted vertically along the *y*-axis according to the time of data collection during the slot-die printing process. Corresponding fits, shown as red lines, are overlaid on each curve. Two characteristic polymer domain peaks evolve during film fabrication (dark orange and light orange arrows). In addition, a peak corresponding to the CoFe_2_O_4_ NPs can be seen at approximately *q*_*y*_ = 0.2 nm^–1^. (b) Radius and distance information on the large polymer domains (D_1_ and R_1_) and small polymer domains (D_2_ and R_2_) taken from the fits. Film formation for the binary hybrid film can be divided into four stages (I–IV): wet film, solvent evaporation, coalescence and microphase separation, and the dry film.

Finally, the evolution of the ternary hybrid film is also investigated. The representative 2D GISAXS data in Figure S3 again show the large wings at high *q*_*y*_ values corresponding now to the presence of both the Ni NPs and the CoFe_2_O_4_ NPs. The correlated roughness is no longer visible due to the NPs influencing the roughness of the film. Similar to the other films, two polymer domain peaks appear and increase in intensity. Furthermore, similar to the binary film, peaks/shoulders corresponding to the two types of NPs contribute to the scattering information between 0.1 nm^–1^ < *q*_*y*_ < 1 nm^–1^. From the fits, it can be clearly seen that the ternary film also shows the four ascribed stages of film formation: wet film, solvent evaporation, rapid microphase separation and coalescence, and the final dry film (Figure 4b).

A comparison of the polymer domain size and center-to-center distance for all three films in the final, dry state is shown in Figure S4. Without NPs, the domain radii and center-to-center distances of the pure DBC film are determined to be (46 ± 2) nm and (137 ± 4) nm for the large domain and (13 ± 2) nm and (75 ± 4) nm for the small polymer domain. Upon addition of 2 wt % CoFe_2_O_4_ NPs, only slight changes in the morphology of the DBC are observed. Thus, while the NPs are incorporated into the DBC film, the NPs do not disturb the thin film morphology during the printing process. For the binary hybrid film, the domain radii and center-to-center distances of the DBC film are determined to be (48 ± 3) nm and (131 ± 4) nm for the large domain and (16 ± 3) nm and (71 ± 4) nm for the small polymer domain. For the ternary hybrid film containing 2 wt % Ni NPs and 2 wt % CoFe_2_O_4_ NPs, larger changes are seen in the film morphology. The domain radii and center-to-center distances of the DBC film are determined to be (60 ± 4) nm and (139 ± 5) nm for the large domain and (17 ± 2) nm and (81 ± 3) nm for the small polymer domain. However, as for the binary hybrid film and confirmed in the AFM and GISAXS investigation, the ternary hybrid DBC film is able to incorporate the relatively low total weight percent of NPs without exhibiting significant morphological changes (Figure 4).

**Figure 4:**
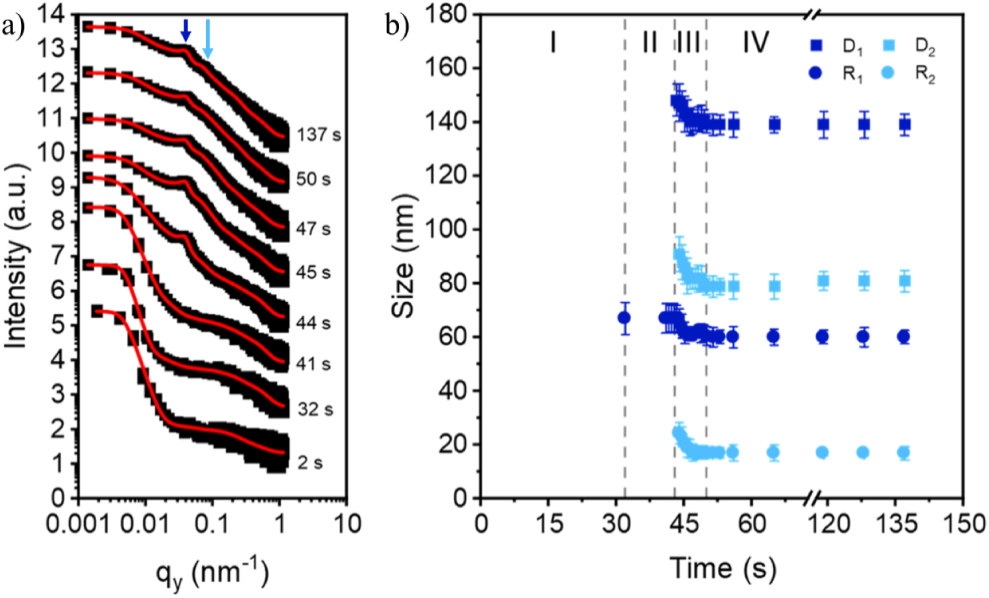
(a) Selected line cuts for the pure PS-*b*-PMMA film containing 2 wt % CoFe_2_O_4_ NPs and 2 wt % Ni NPs taken from the 2D GISAXS data. The curves are shifted vertically along the *y*-axis according to the time of data collection during the slot-die printing process. Corresponding fits, shown as red lines, are overlaid on each curve. Two characteristic polymer domain peaks evolve during film fabrication (dark blue and light blue arrows), and the minor contributions from the CoFe_2_O_4_ and Ni NPs can be seen at large *q*_*y*_ values between approximately *q*_*y*_ = 0.1 nm^–1^ and 1 nm^–1^. (b) Radius and distance information on the large polymer domains (D_1_ and R_1_) and small polymer domains (D_2_ and R_2_) taken from the fits. Film formation for the ternary 1hybrid film can also be divided into four stages (I–IV): wet film, solvent evaporation, coalescence and microphase separation, and the dry film.

### 3.3 Magnetic Properties

#### 3.3.1 Temperature-Dependence and Magnetic Interactions

The in-plane (IP) magnetic behavior of the binary hybrid film and the ternary hybrid film along the direction of printing is investigated at five different temperatures, 5, 50, 100, 200, and 300 K, to examine the influence of temperature on the magnetic properties. The magnetic hysteresis curves for the binary hybrid film containing 2 wt % CoFe_2_O_4_ NPs measured between −5 kOe and 5 kOe are shown in Figure 5a, where the magnetization *M* is expressed per unit volume as calculated from the film dimensions. At 300 K, the film containing only CoFe_2_O_4_ NPs shows the expected ferrimagnetic behavior with the binary hybrid film having a saturation magnetization *M*_s_ of 0.6 ± 0.1 emu cm^–3^, remanence *M*_r_ of 0.15 ± 0.01 emu cm^–3^, and a coercivity *H*_c_ of 220 ± 1 Oe. As the temperature is decreased, the thermal fluctuations of the magnetic domains are reduced, and the strength of the magnetic field that is required to flip the domains increases.^57,58^ This behavior leads to the observed increase in *M*_r_ and *H*_c_ with decreasing temperature in the binary hybrid film, while the *M*_s_ remains relatively unchanged, around 0.7 emu cm^–3^, as shown in Figure 6. The remanence increases to 0.22 ± 0.01 emu cm^–3^ at 200 K and linearly increases further to 0.27 ± 0.01 emu cm^–3^, 0.29 ± 0.01 emu cm^–3^, and 0.56 ± 0.3 emu cm^–3^ at 100, 50, and 5 K. The increase in coercivity is also observed to be approximately linear, increasing to 318 ± 1 Oe at 200 K and further to 432 ± 5 Oe, 487 ± 4 Oe, and 653 ± 36 Oe at 100, 50, and 5 K.

**Figure 5:**
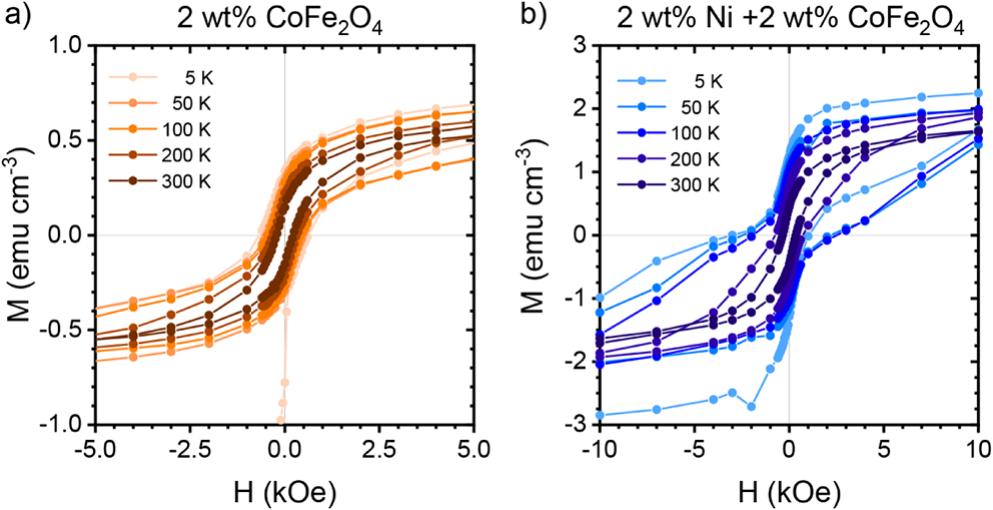
Temperature-dependent magnetic hysteresis curves for the (a) binary hybrid film and the (b) ternary hybrid film measured at 5, 50, 100, 200, and 300 K. For ease of representation, the curves are plotted between −5 kOe and 5 kOe for the binary hybrid film and between −10 kOe and 10 kOe for the ternary hybrid film.

**Figure 6:**
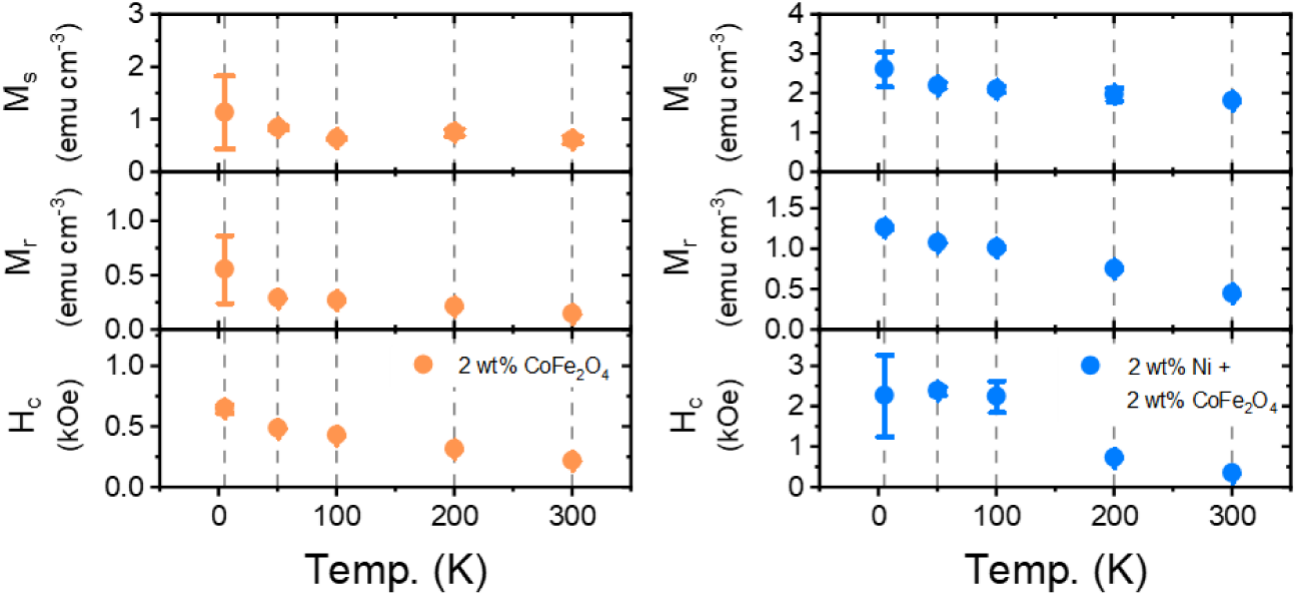
Saturation magnetization (*M*_s_), remanence (*M*_r_), and coercivity (*H*_c_) as a function of temperature for the binary hybrid film (orange) and the ternary hybrid film (blue). The data is extracted from the corresponding magnetization curves.

The magnetic hysteresis curves for the ternary hybrid film containing 2 wt % CoFe_2_O_4_ NPs and 2 wt % Ni NPs measured at the same temperatures are shown in Figure 5b. With the addition of the Ni NPs, the sensitivity of the ternary hybrid film is enhanced, and *M*_s_ increases to 1.8 ± 0.1 emu cm^–3^ at 300 K. An increase in *M*_r_ and *H*_c_ is also observed, with *M*_r_ and *H*_c_ values of 0.45 ± 0.01 emu cm-3 and 356 ± 1 Oe. Similar to the magnetic properties of the binary hybrid, *M*_r_ and *H*_c_ for the ternary hybrid film increase as the temperature is decreased, while *M*_s_ remains relatively unchanged around 2 emu cm^–3^, as shown in Figure 6. The remanence increases linearly to 0.76 ± 0.01 emu cm^–3^, 1.02 ± 0.02 emu cm^–3^, 1.08 ± 0.02 emu cm^–3^, and 1.27 ± 0.02 emu cm^–3^ for 200, 100, 50, and 5 K. In the case of the coercivity, an initial increase to 737 ± 15 Oe is observed at 200 K. For temperatures at or below 100 K, the coercivity jumps to 2250 ± 390 Oe, 2390 ± 100 Oe, and 2280 ± 1000 Oe for 100, 50, and 5 K. Overall, it is observed that the *H*_c_ values of both the binary and ternary films are less than what was observed in our previous study of CoFe_2_O_4_ NPs pellets.^50^ This may be due to the low mass fraction of NPs in the films. A similar trend has been observed in films of Fe NPs in a PMMA matrix, where the *H*_c_ value is reduced upon decreasing the total mass of Fe in the film below a critical threshold.^59^ Furthermore, the increase in the coercivity of the ternary system at and below 100 K is accompanied by a noticeable change in the shape of the hysteresis loops below 200 K. As the temperature decreases, the curves show a more pronounced double-step hysteresis loop, a so-called necking behavior around zero magnetic field. As discussed above in the case of the binary hybrid film, the observed increase of *H*_c_ in the ternary film as temperature decreases is due to the larger field required at lower temperatures to reverse magnetization. The double-step hysteresis loops, recorded after zero-field cooling, at low temperature in the ternary films, are due to the superposition of the square hysteresis loops of the hard CoFe_2_O_4_ NPs and the sharp loops of the soft Ni NPs.^50^ Thus, this necking behavior in the ternary film corresponds to the two distinct switching fields of the Ni NPs and CoFe_2_O_4_ NPs, suggesting that the two magnetic phases are not exchange-coupled.^60,61^ To confirm this, the distinct switching fields of the two magnetic phases are identified by plotting the dM/dH vs H curves for the ternary hybrid film at the measured temperatures, as shown in Figure S5.^62,63^ At 300 K, a single peak is observed as the switching fields of the two magnetic phases overlap. As the temperature is decreased, a shoulder appears and develops into a broad peak at large applied field values, and the two distinct switching fields of the soft Ni NPs and hard CoFe_2_O_4_ NPs are clearly seen. While the necking behavior can be attributed to the distinct switching fields of the hard and soft NPs in the system, this alone does not explain the enhancement of the coercivity of the ternary system in comparison to the system containing only the hard CoFe_2_O_4_ NPs. This coercivity enhancement could be due to the presence of different NP populations in the systems, such as aggregates of soft Ni NPs and hard CoFe_2_O_4_ NPs that are coupled through additional dipolar interactions.^17,18^ The large uncertainty in the coercivity at 5 K arises from the observed difference in the coercive fields of the demagnetization and magnetization processes, as seen in the hysteresis curve in Figure 5b.

#### 3.3.2 Perpendicular Magnetic Anisotropy

The impact of the direction of the magnetic field on the resulting magnetic behavior of the printed hybrid films is examined by measuring the out-of-plane (OOP) magnetization of the hybrid films. For the OOP plane measurements, the magnetic field is applied perpendicular to film plane again at five different temperatures, 5, 50, 100, 200, and 300 K. A schematic of the difference between the in-plane and out-of-plane orientations is shown in Figure S6. The difference in the IP and OOP behavior of the binary hybrid film containing 2 wt % CoFe_2_O_4_ NPs measured at 100 and 300 K is clearly observed from the magnetic hysteresis curves shown in Figure 7a and Figure 7b. For both temperatures, the OOP measurements are characterized by higher *M*_s_, and lower *M*_r_ and *H*_c_ as compared to the IP measurements. At 100 K, *M*_s_ increases from 0.6 ± 0.1 emu cm^–3^ for the in-plane orientation to 2.0 ± 0.1 emu cm^–3^ for the out-of-plane orientation, while *M*_r_ decreases from 0.27 ± 0.01 emu cm^–3^ to 0.07 ± 0.01 emu cm^–3^, and *H*_c_ decreases from 432 ± 5 Oe to 102 ± 15 Oe. At 300 K, *M*_s_ increases from 0.6 ± 0.1 emu cm^–3^ for the in-plane orientation to 2.3 ± 0.1 emu cm^–3^ for the out-of-plane orientation, while *M*_r_ decreases from 0.15 ± 0.01 emu cm^–3^ to 0.07 ± 0.1 emu cm^–3^, and *H*_c_ decreases from 220 ± 1 Oe to 82 ± 10 Oe.

**Figure 7:**
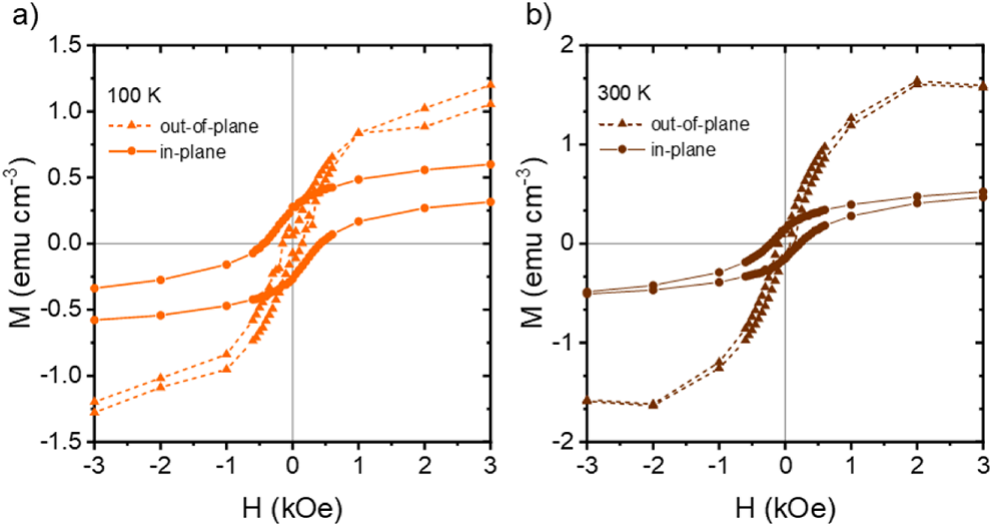
In-plane and out-of-plane magnetic hysteresis curves for the binary hybrid film containing 2 wt % CoFe_2_O_4_ NPs measured at (a) 100 K and (b) 300 K. At both temperatures, the out-of-plane data demonstrate an increased saturation magnetization and a reduced coercivity compared to the in-plane data.

Similarly, in Figure 8a,b, the difference in the IP and OOP magnetic behavior for the ternary hybrid film containing 2 wt % Ni NPs and 2 wt % CoFe_2_O_4_ NPs at 300 and 100 K can be seen. Again, the OOP measurements of the ternary hybrid film show an increased *M*_s_ and a lower *M*_r_ and *H*_c_ as compared to the IP measurements. At 100 K, the OOP *M*_s_ increases to 3.3 ± 0.1 emu cm^–3^, and the *M*_r_ and *H*_c_ decrease to 0.62 ± 0.01 emu cm^–3^ and 365 ± 10 Oe. At 300 K, *M*_s_ is measured to be 3.1 ± 0.1 emu cm^–3^, and the *M*_r_ and *H*_c_ are 0.23 ± 0.01 emu cm^–3^ and 111 ± 2 Oe. A full comparison of the IP and OOP measurements for the binary and ternary hybrid films at all five investigated temperatures can be seen in Figure S7.

**Figure 8:**
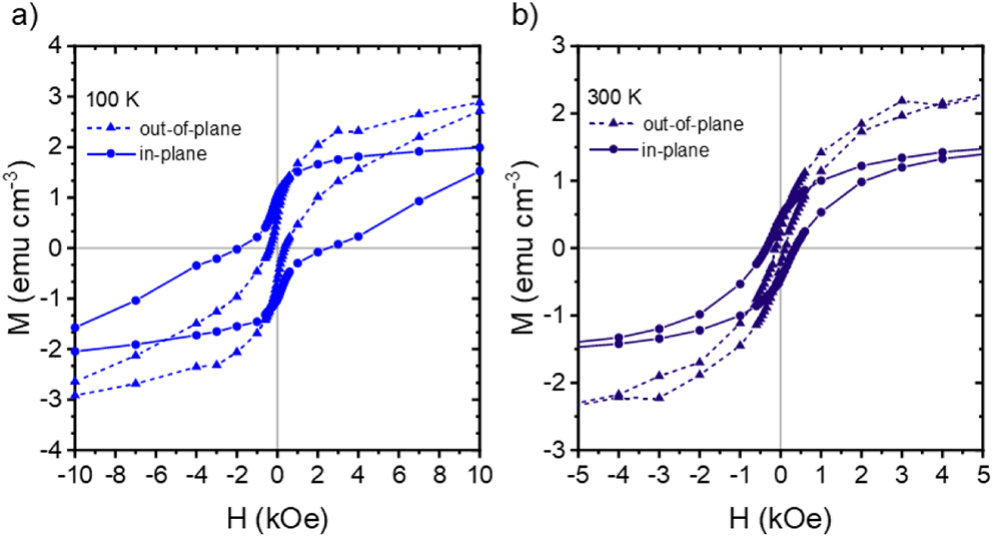
In-plane and out-of-plane magnetic hysteresis curves for the ternary hybrid film containing 2 wt % CoFe_2_O_4_ NPs and 2 wt % Ni NPs measured at (a) 100 K and (b) 300 K. At both temperatures, the out-of-plane data demonstrate an increased saturation magnetization and a reduced coercivity in comparison to the in-plane data.

The uniaxial magnetic anisotropy observed in both the binary hybrid film and the ternary hybrid film likely results from the presence and preferential orientation of NP aggregates or assemblies inside the DBC films.^64–68^ In the case of the binary hybrid DBC film containing 2 wt % CoFe_2_O_4_ NPs, the increase in the saturation magnetization *M*_s_ and decrease in the remanence *M*_r_ and coercivity *H*_c_ for the OOP magnetic measurements suggest that the NPs are oriented vertically along the depth of the film inside the NP domains with the easy axis perpendicular to the film surface.^69–71^ This explanation can be extended to the ternary hybrid thin films containing 2 wt % Ni NPs and 2 wt % CoFe_2_O_4_ NPs. Further analysis of the differences between the IP and OOP measurements, such as the determination of the demagnetization field, could provide more insight into the observed behavior.

## 4 Conclusions

In this work, hybrid soft/hard DBC-NP composites are fabricated through a slot-die printing technique and examined for use as unique ESM materials. To evaluate the impact of the NPs on the thin film morphology, the film formation is tracked *in situ* utilizing GISAXS. The results are compared to those of a binary hybrid film containing only the hard magnetic material and to those of a pure DBC film containing no NPs. It is observed that for such low NP concentrations, the PS-*b*-PMMA film morphology is relatively undisturbed upon the addition of the magnetic NPs, with only small changes observed in the size and distribution of the polymer domains. This finding confirms the robustness of the UHMW DBC film as a scaffold for the inorganic NPs. All of the investigated films show four stages of film formation: the wet film, solvent evaporation, rapid coalescence and microphase separation, and the final dry film. The magnetic properties of the binary and ternary hybrid films are investigated using a SQUID magnetometer with the magnetic field oriented along the film plane, in-plane, as well as perpendicular to the film plane, out-of-plane. In the in-plane direction, the binary hybrid film containing only CoFe_2_O_4_ NPs has a typical temperature-dependent magnetic behavior of a ferrimagnetic material with a decrease in temperature corresponding to an increase in *M*_r_ and *H*_c_ due to the reduced thermal fluctuations in the magnetic domains of the NPs. Upon addition of Ni NPs, the ternary film shows an increase in *M*_s_ as well as an increase in *M*_r_ and *H*_c_ as the temperature is decreased. Upon investigation of the switching field distribution, it is evident that soft and hard phases do not exhibit exchange coupling, as observed by the two-phase hysteresis loops. However, the increase of *H*_c_ in the ternary film compared to the binary film is attributed to dipolar interactions between the two magnetic phases. Compared to the in-plane magnetic behavior, the out-of-plane magnetic measurements for both films exhibit an increased *M*_s_ and decreased *M*_r_ and *H*_c_ for all investigated temperatures due to the orientation of the magnetic field along the easy axis of the NPs inside the films. A focused analysis of the temperature dependence, through comparison of the data presented in this study to the magnetic behavior of the NPs without the influence of the DBC template, would be necessary to fully understand the magnetic response of the hybrid films. Thus, the fabrication of ternary DBC-NP composites containing both soft and hard magnetic NPs enables the manufacture of magnetically coupled composites through a scalable fabrication process.

## Supplementary Material



## Notes

Additional experimental details, 2D GISAXS images, comparison of polymer domain characteristics, switching field distribution, and in-plane and out-of-plane magnetic properties of hybrid films (PDF)
