# Supplementary material for: Structural Evolution of Printed Ternary Magnetic Hybrid Thin Films Containing Soft and Hard Magnetic Nanoparticles for Coupled Composites
Source: ACS Appl Mater Interfaces. 2025 Nov 25;17(49):67132–40. doi: 10.1021/acsami.5c16986 (PMC12874353; doi:10.1021/acsami.5c16986)
Supplement: Supplementary file 1 [file am5c16986_si_001.pdf]

Supporting Information

Structural evolution of printed ternary magnetic hybrid thin films containing soft and hard magnetic nanoparticles for coupled composites

Christopher R. Everett[1], Guangjiu Pan[1], Manuel A. Reus[1], David P. Kosbahn[1], Aidin Lak[2], Frank Hartmann[3], Martin Bitsch[3], Markus Gallei[3,4], Matthias Opel[5], Matthias Schwartzkopf[6], Peter Müller-Buschbaum[1,]*

[1] Technical University of Munich, TUM School of Natural Sciences, Department of Physics, Chair for Functional Materials, James-Franck-Str. 1, 85748 Garching, Germany

[2] Institute for Electrical Measurement Science and Fundamental Electrical Engineering and Laboratory for Emerging Nanometrology (LENA), TU Braunschweig, Hans-Sommer-Str. 66, 38106 Braunschweig, Germany

[3]Chair in Polymer Chemistry, Saarland University, Campus C4 2, 66123 Saarbrücken, Germany

[4] Saarene, Saarland Center for Energy Materials and Sustainability, Campus C4 2, 66123 Saarbrücken, Germany

[5] Walther-Meissner-Institut, Bayerische Akademie der Wissenschaften, Walther-Meissner-Str. 8, 85748 Garching, Germany

[6] Deutsches Elektronen-Synchrotron (DESY), Notkestr. 85, 22607 Hamburg, Germany

* Corresponding author Email: muellerb@ph.tum.de

In Situ GISAXS Experimental Parameters

The X-ray beam wavelength was 0.1048 nm, and a sample-detector distance (SDD) of 4013 mm was chosen. The incidence angle was set to 0.4° for all measurements, as this angle is above the critical angle of the DBC and NPs. The scattered signal from the sample was recorded on a two-dimensional detector (Pilatus 2M, Dectis) with a pixel size of 172 μm × 172 μm. The exposure time was set to 0.3 s, and each captured image was taken at a new sample position to avoid overexposing the hybrid films.

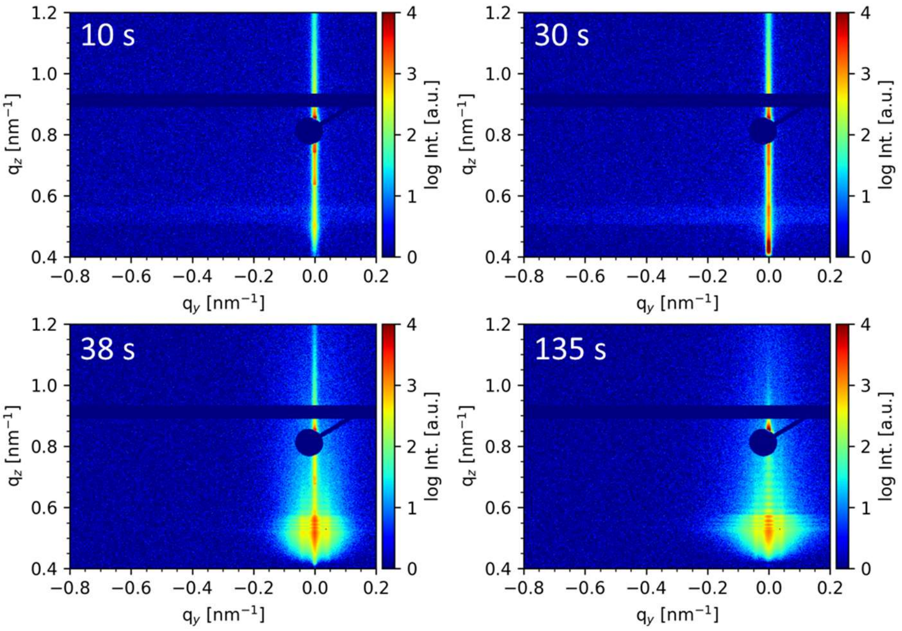

Figure S1: Representative 2D GISAXS data for the PS-*b*-PMMA film with no NPs. To prevent oversaturation of the detector, the specular beam was blocked with a circular beam stop. As time increases, the evolution of the DBC film morphology can be observed.

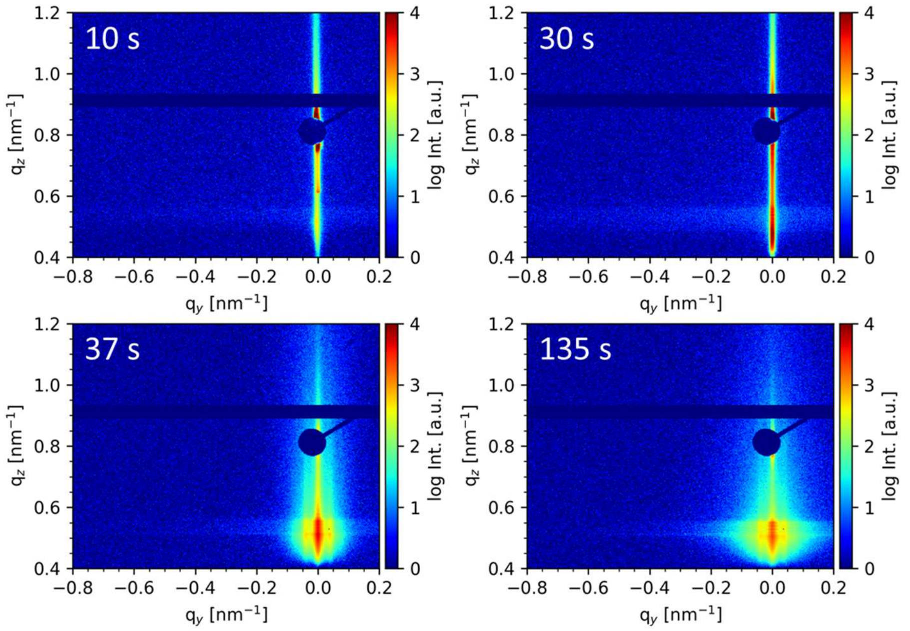

Figure S2: Representative 2D GISAXS data for the PS-*b*-PMMA film containing 2 wt% $CoFe_2O_4$ NPs. To prevent oversaturation of the detector, the specular beam was blocked with a circular beam stop. As time increases, the evolution of the DBC film morphology can be observed. The appearance of weak 'band-like' scattering features extending from near the Yoneda region to large q_y values is attributed to the NPs.

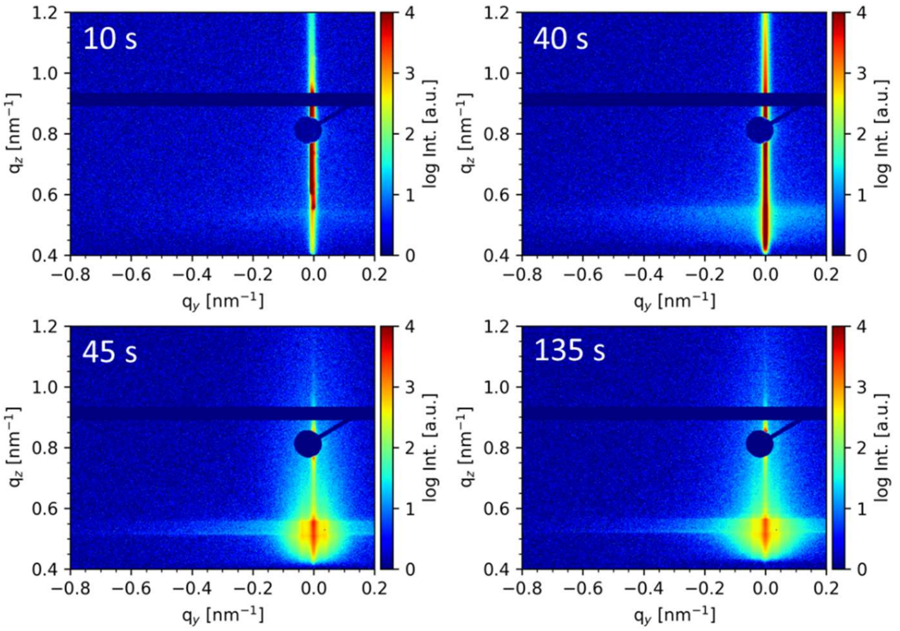

Figure S3: Representative 2D GISAXS data for the PS-*b*-PMMA film containing both 2 wt% $CoFe_2O_4$ NPs and 2 wt% Ni NPs. To prevent oversaturation of the detector, the specular beam was blocked with a circular beam stop. As time increases, the evolution of the DBC film morphology can be observed. The appearance of 'band-like' scattering features extending from near the Yoneda region to large q_y values is attributed to the NPs.

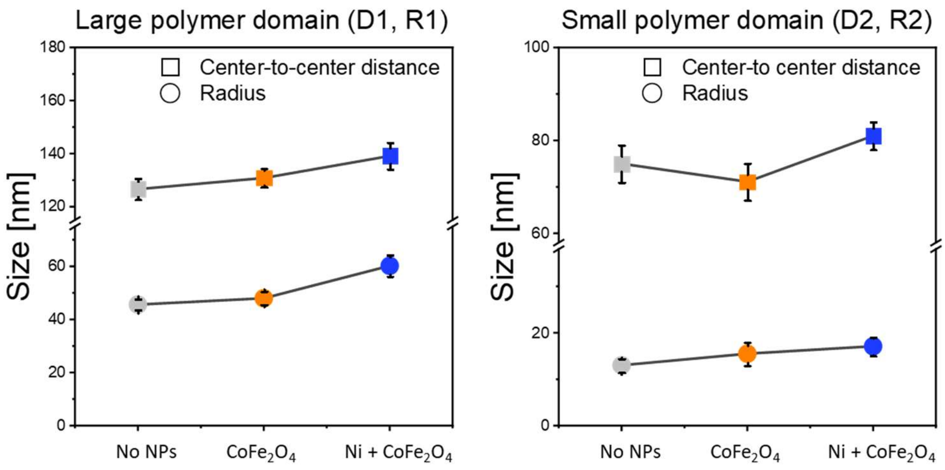

Figure S4: Comparison of the domain size and center-to-center distance of the polymer domains for each investigated film at the end of the *in situ* investigation (stage IV, dry film) for (left) the large polymer domain and (right) the small polymer domain. With the addition of NPs, polymer domain sizes and center-to-center distances increase, with more pronounced changes observed for the large polymer domain and slight changes seen in the small polymer domain.

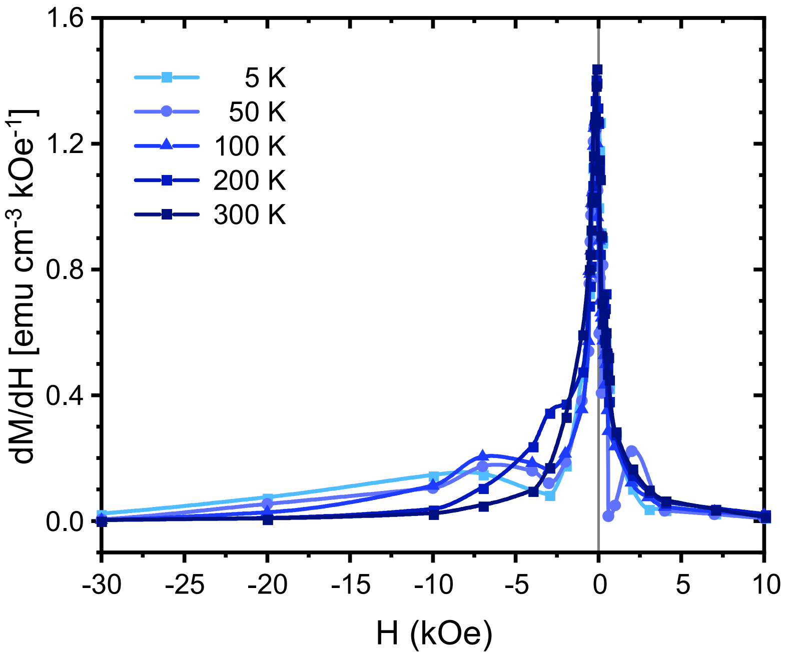

Figure S5: Switching field distribution curves (dM/dH) of the demagnetization curves for the ternary hybrid thin film containing 2 wt% Ni NPs and 2 wt% $CoFe_2O_4$ NPs. At 300 K, a single peak is observed as the switching fields of the two magnetic phases overlap. As the temperature decreases, two peaks are observed corresponding to the distinct switching fields of the soft Ni NPs and the hard $CoFe_2O_4$ NPs.

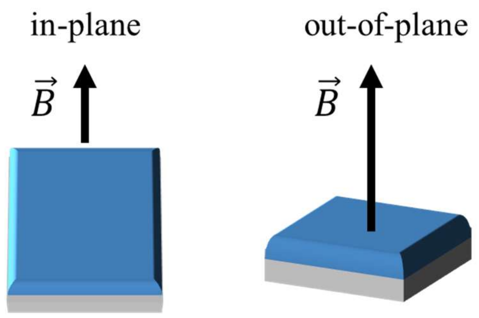

Figure S6: Schematic detailing the direction of the applied magnetic field in relation to the thin film for the in-plane and out-of-plane magnetic measurements. The in-plane orientation refers to the situation where the applied magnetic field lies parallel to the plane of the thin film, whereas the out-of-plane orientation refers to the situation where the applied magnetic field is perpendicular to the plane of the thin film. The applied magnetic field is therefore rotated by 90° between the in-plane and out-of-plane orientations.

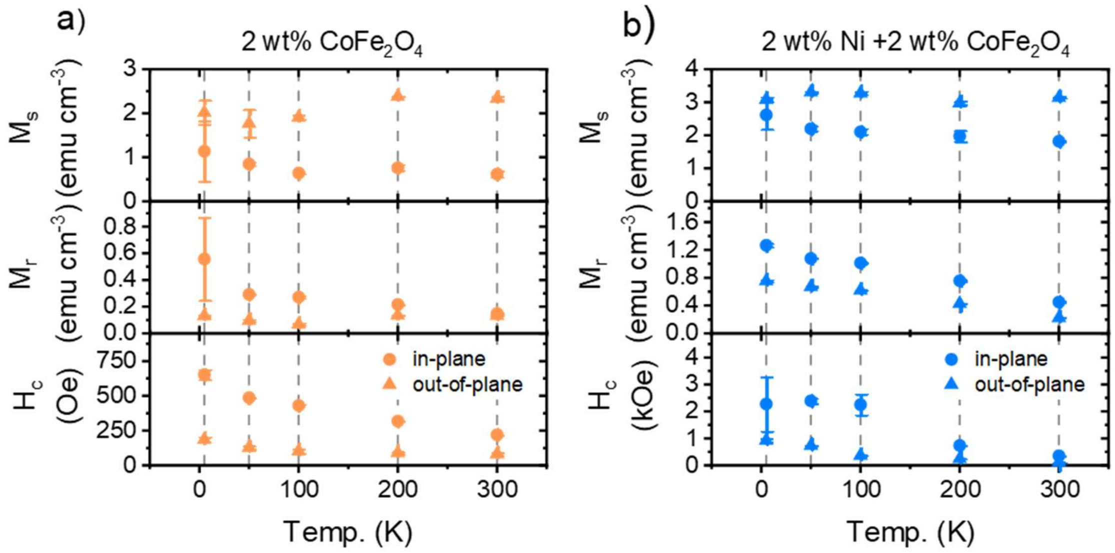

Figure S7. (a) Comparison of the saturation magnetization (M_s), remanence (M_r), and coercivity (H_c) between the in-plane magnetic measurements (orange circles) and out-of-plane magnetic measurements (orange triangles) as a function of temperature for the binary hybrid film containing 2 wt% $CoFe_2O_4$ NPs. (b) Comparison of the saturation magnetization (M_s), remanence (M_r), and coercivity (H_c) between the in-plane magnetic measurements (blue circles) and out-of-plane magnetic measurements (blue triangles) as a function of temperature for the ternary hybrid film containing 2 wt% $CoFe_2O_4$ NPs and 2 wt% Ni NPs. For all temperatures, the out-of-plane measurements of both films show increased M_s and decreased H_c.
